# Childhood cancer in the south Asian population of England (1990–1992)

**DOI:** 10.1054/bjoc.2001.1775

**Published:** 2001-05

**Authors:** C Cummins, H Winter, R Maric, K K Cheng, P Silcocks, C Varghese, G Batlle

**Affiliations:** Department of Public Health and Epidemiology, The University of Birmingham, Edgbaston, Birmingham, B15 2TT, UK; Institute of Child Health, Clinical Research Block, Whittall Street, Birmingham, B4 6NH; Trent Cancer Registry, Weston Park Hospital, Whitham Road, Sheffield, S10 2SJ, UK; Trent Institute for Health Services Research, Room B39, Queen's Medical Centre, University of Nottingham, Nottingham, NG7 2UH; Yorkshire Cancer Registry, Centre for Cancer Research, University of Leeds, Arthington House, Cookridge Hospital, Leeds, LS16 6QB, UK; Cancer Epidemiology and Clinical Research Division, Regional Cancer Centre, Trivandrum, India 695011; West Midlands Cancer Intelligence Unit, Public Health Building, The University of Birmingham, Edgbaston, Birmingham, B15 2TT, UK

**Keywords:** child, cancer incidence, south Asian, migrants, England

## Abstract

Cancer incidence in 1990–92 among English south Asian (residents with ethnic origins in India, Pakistan or Bangladesh) and non-south Asian children is compared. Standardized incidence ratios show significant overall excesses in south Asians (131), largely due to higher rates in south Asian boys, and specific excesses for leukaemia (141), lymphoid leukaemia (141), lymphoma (172) and hepatic tumours (375). Aetiological investigation is required. © 2001 Cancer Research Campaign http://www.bjcancer.com


*British Journal of Cancer* (2001) **84**(9), 1215–1218
© 2001 Cancer Research Campaign
doi: 10.1054/ bjoc.2001.1775, available online at http://www.idealibrary.com on IDEAL®

http://www.bjcancer.com

Short Communication

### Childhood cancer in the south Asian population of England (1990–1992)

**C Cummins[1,2], H Winter[1], R Maric[1], KK Cheng[1], P Silcocks[3,4], C Varghese[5,6] and G Batlle[7]**

[1]Department of Public Health and Epidemiology, The University of Birmingham, Edgbaston, Birmingham, B15 2TT, UK; [2]Institute of Child Health, Clinical Research Block, Whittall Street, Birmingham, B4 6NH; [3]Trent Cancer Registry, Weston Park Hospital, Whitham Road, Sheffield, S10 2SJ, UK; [4]Trent Institute for Health Services Research, Room B39, Queen's Medical Centre, University of Nottingham, Nottingham, NG7 2UH; [5]Yorkshire Cancer Registry, Centre for Cancer Research, University of Leeds, Arthington House, Cookridge Hospital, Leeds, LS16 6QB, UK; [6]Cancer Epidemiology and Clinical Research Division, Regional Cancer Centre, Trivandrum, India 695011; [7]West Midlands Cancer Intelligence Unit, Public Health Building, The University of Birmingham, Edgbaston, Birmingham, B15 2TT, UK

**Summary** Cancer incidence in 1990–92 among English south Asian (residents with ethnic origins in India, Pakistan or Bangladesh) and non-south Asian children is compared. Standardized incidence ratios show significant overall excesses in south Asians (131), largely due to higher rates in south Asian boys, and specific excesses for leukaemia (141), lymphoid leukaemia (141), lymphoma (172) and hepatic tumours (375). Aetiological investigation is required. © 2001 Cancer Research Campaign http://www.bjcancer.com

**Keywords**: child; cancer incidence; south Asian; migrants; England

There are more than 1.4 million residents in England with ethnic origins in the Indian sub-continent, representing approximately 3.0% of the total population and 5.4% of the population aged 0–14 (OPCS, 1993). Despite substantial religious and cultural heterogeneity within the group as a whole (referred to as south Asian in this paper), differences from other ethnic groups have been demonstrated for a wide range of health outcomes (Soni Raleigh et al, 1990; Wild and McKeigue, 1997; Mather et al, 1998). Previous studies on childhood cancer in the south Asian population have either been limited to regional geographical areas (Muir et al, 1992, 1995; Powell et al, 1994, 1995; Varghese et al, 1996) or have not reported incidence or mortality rates (Stiller et al, 1991; Swerdlow et al, 1995) through lack of population denominator data. This study combined numerator data from 4 regional cancer registries with denominator data from the 1991 census, incorporating approximately 80% of the resident south Asian population, to derive the first near national sex-specific estimates of childhood cancer incidence in the south Asian population of England.

The study methods have been described in detail elsewhere (Cummins et al, 1999; Winter et al, 1999). The data set consisted of cases of incident cancer (ICD9: 140–208) in children aged 0–14 years registered between 1990 and 1992, in areas covered by Thames, Trent, West Midlands and Yorkshire cancer registries. The list of cancer registrations was run against the Nam Pehchan computer software package (Bradford Health Authority) to produce a list of matches against the program's dictionary of south Asian names and then subjected to a range of further computer and visual inspection methods to classify each case as either south Asian or non south Asian. Denominator data allowing the calculation of incidence rates by ethnic group was provided by the 1991 census (OPCS, 1993). For the purpose of the study, a south Asian case was defined as a person whose name was identified as consistent with an ethnic origin in peoples indigenous to India, Pakistan or Bangladesh thus including migrants with these ethnic origins from East Africa and elsewhere, while the south Asian denominator consisted of the population with self-ascribed ethnicity to these countries in the 1991 census. All cases aged 0 –14 were classified according to the International Classification of Childhood Cancer (Kramarova and Stilker, 1996). Incidence rates directly standardized to the world standard population and standardized incidence ratios with 95% confidence intervals were calculated for each of the main diagnostic groups. The standardized incidence ratios were calculated as the ratio between the observed and expected number of south Asian cases using the non-south Asian population as the standard population. Confidence intervals for the standardized incidence ratios were based on exact 95% confidence intervals for the expectation of a Poisson distribution according to the number of observed cases where the number of south Asian cases was less than or equal to 60 and on the Normal approximation to the Poisson distribution where the number of south Asian cases exceeded 60 (Esteve et al, 1994).

The Nam Pehchan computer program identified 203 south Asian names from the 1819 cases considered in total. After visual inspection, 160 program positives were confirmed as south Asian and 16 program negatives were also classified as south Asian, yielding a total of 176 south Asian cases (9.7%) in the final classification. Age-standardized incidence rates (ASR) and standardized incidence ratios (SIR%) for the main diagnostic groups are presented in Tables 1 and 2. Overall incidence rates were higher in south Asians (ASR 15.0) than non-south Asians (ASR 11.5) and higher in males than females for both south Asians (ASR 18.7 cf. 11.1) and non-south Asians (ASR 12.6 cf. 10.3). Standardized incidence ratios indicated a significant excess of south Asian cases for leukaemia (SIR 141), lymphoid leukaemia (SIR 141), lymphoma (SIR 172) and hepatic tumours (SIR 375) in both sexes combined, and for lymphoma (SIR 210), retinoblastoma (SIR 384)

*Received 9 January 2001*
*Revised 19 February 2001*
*Accepted 19 February 2001*

*Correspondence to:* C Cummins

**Table 1** Age-standardized incidence rates per 100 000 person-years (1990–92)

| | South Asian | | | Non-south Asian | | |
|---|---|---|---|---|---|---|
| **Sex** | ***n*** | **Rate** | **95%CI** | ***n*** | **Rate** | **95%CI** |
| Male | 112 | 18.7 | 15.1–22.2 | 930 | 12.6 | 11.8–13.5 |
| Female | 64 | 11.1 | 8.3–13.9 | 713 | 10.3 | 9.5–11.0 |
| Total | 176 | 15.0 | 12.7–17.3 | 1643 | 11.5 | 10.9–12.1 |

**Table 2** Age-standardized incidence rates per $10^5$ person-years and standardized incidence ratios (1990–92)

| | | South Asian | | Non-south Asian | | SIR | |
|---|---|---|---|---|---|---|---|
| **Diagnostic group** | **Sex** | ***n*** | **Rate** | ***n*** | **Rate** | **Ratio** | **95%CI** |
| I. leukaemia | m | 36 | 6.1 | 326 | 4.5 | 137 | 95–189 |
| | f | 28 | 4.9 | 236 | 3.4 | 146 | 97–212 |
| | mf | 64 | 5.5 | 562 | 4.0 | 141* | 108–180 |
| Ia. lymphoid leukaemia | m | 30 | 5.2 | 265 | 3.6 | 140 | 94–201 |
| | f | 21 | 3.7 | 184 | 2.7 | 141 | 87–217 |
| | mf | 51 | 4.5 | 449 | 3.2 | 141* | 104–186 |
| II. lymphomas | m | 23 | 3.8 | 128 | 1.7 | 210* | 133–316 |
| | f | 4 | 0.7 | 57 | 0.8 | 84 | 22–215 |
| | mf | 27 | 2.3 | 185 | 1.3 | 172* | 113–250 |
| IIa. Hodgkin’s disease | m | 9 | 1.5 | 50 | 0.6 | 207 | 94–392 |
| | f | 1 | 0.2 | 22 | 0.3 | 52 | 1–290 |
| | mf | 10 | 0.8 | 72 | 0.5 | 159 | 76–293 |
| IIb. non-Hodgkin’s lymphoma | m | 6 | 0.9 | 38 | 0.5 | 184 | 67–400 |
| | f | 1 | 0.2 | 19 | 0.3 | 65 | 1–364 |
| | mf | 7 | 0.6 | 57 | 0.4 | 146 | 58–301 |
| III-XII. other groups | m | 53 | 8.8 | 476 | 6.5 | 136* | 102–179 |
| | f | 32 | 5.6 | 420 | 6.0 | 93 | 63–132 |
| | mf | 85 | 7.2 | 896 | 6.3 | 116 | 92–144 |
| III. central nervous system | m | 19 | 3.0 | 178 | 2.4 | 127 | 76–199 |
| | f | 11 | 1.8 | 175 | 2.5 | 76 | 37–137 |
| | mf | 30 | 2.4 | 353 | 2.4 | 102 | 68–146 |
| IV. sympathetic nervous system | m | 4 | 0.8 | 82 | 1.2 | 64 | 17–165 |
| | f | 5 | 0.9 | 53 | 0.8 | 121 | 39–283 |
| | mf | 9 | 0.8 | 135 | 1.0 | 87 | 39–165 |
| V. retinoblastoma | m | 7 | 1.3 | 24 | 0.3 | 384* | 153–790 |
| | f | 0 | 0.0 | 23 | 0.3 | 0 | 0–170 |
| | mf | 7 | 0.7 | 47 | 0.3 | 195 | 78–402 |
| VI. renal tumours | m | 2 | 0.3 | 40 | 0.6 | 63 | 7–230 |
| | f | 2 | 0.4 | 40 | 0.6 | 65 | 7–235 |
| | mf | 4 | 0.4 | 80 | 0.6 | 64 | 17–165 |
| VII. hepatic tumours | m | 2 | 0.4 | 10 | 0.1 | 242 | 29–874 |
| | f | 2 | 0.3 | 3 | 0.0 | 837 | 100–3021 |
| | mf | 4 | 0.3 | 13 | 0.1 | 375* | 102–961 |
| VIII. malignant bone tumours | m | 7 | 1.0 | 45 | 0.6 | 177 | 70–365 |
| | f | 2 | 0.4 | 41 | 0.6 | 56 | 6–204 |
| | mf | 9 | 0.7 | 86 | 0.6 | 120 | 54–228 |
| IX. soft-tissue sarcomas | m | 6 | 1.0 | 56 | 0.8 | 132 | 48–288 |
| | f | 5 | 0.9 | 45 | 0.6 | 136 | 44–317 |
| | mf | 11 | 0.9 | 101 | 0.7 | 134 | 66–239 |
| X. germ cell | m | 3 | 0.5 | 16 | 0.2 | 233 | 48–681 |
| | f | 3 | 0.5 | 17 | 0.2 | 211 | 43–618 |
| | mf | 6 | 0.5 | 33 | 0.2 | 222 | 81–483 |
| XI. carcinoma | m | 0 | 0.0 | 20 | 0.3 | 0 | 0–173 |
| | f | 1 | 0.2 | 19 | 0.3 | 61 | 1–342 |
| | mf | 1 | 0.1 | 39 | 0.3 | 30 | 0–166 |
| XII. other/unspecified | m | 3 | 0.4 | 5 | 0.1 | 727* | 150–2125 |
| | f | 1 | 0.2 | 4 | 0.1 | 296 | 8–1647 |
| | mf | 4 | 0.3 | 9 | 0.1 | 531* | 144–1360 |
| total | m | 112 | 18.7 | 930 | 12.6 | 147* | 120–177 |
| | f | 64 | 11.1 | 713 | 10.3 | 110 | 84–141 |
| | mf | 176 | 15.0 | 1643 | 11.5 | 131* | 112–152 |

*95%CI for standardized incidence ratio (SIR) does not include 100.

 *© 2001 Cancer Research Campaign*

**Table 3** Standardized incidence ratios (SIR) by main diagnostic group and 5-year age group

| Diagnostic group | | Male | | | Female | | | Total | | |
|---|---|---|---|---|---|---|---|---|---|---|
| | Age group | *n* | SIR | 95%CI | *n* | SIR | 95%CI | *n* | SIR | 95%CI |
| Leukaemia | 0–4 | 18 | 142 | 84–225 | 12 | 122 | 62–213 | 30 | 133 | 89–191 |
| | 5–9 | 10 | 109 | 52–200 | 10 | 171 | 81–315 | 20 | 133 | 80–205 |
| | 10–14 | 8 | 177 | 76–350 | 6 | 175 | 64–382 | 14 | 177 | 96–297 |
| | 0–14 | 36 | 137 | 95–189 | 28 | 146 | 97–212 | 64 | 141* | 108–180 |
| Lymphomas | 0–4 | 9 | 504* | 230–957 | 1 | 70 | 2–391 | 10 | 311* | 149–573 |
| | 5–9 | 7 | 160 | 64–329 | 1 | 94 | 2–524 | 8 | 146 | 63–289 |
| | 10–14 | 7 | 146 | 58–302 | 2 | 88 | 10–317 | 9 | 128 | 58–243 |
| | 0–4 | 23 | 210* | 133–316 | 4 | 84 | 22–215 | 27 | 172* | 113–250 |
| Other groups | 0–4 | 22 | 134 | 83–203 | 13 | 88 | 46–151 | 35 | 112 | 78–157 |
| | 5–9 | 15 | 127 | 70–209 | 8 | 88 | 38–175 | 23 | 110 | 69–166 |
| | 10–14 | 16 | 151 | 86–245 | 11 | 103 | 51–184 | 27 | 127 | 83–185 |
| | 0–14 | 53 | 136* | 102–179 | 32 | 93 | 63–132 | 85 | 116 | 92–144 |
| Total | 0–4 | 49 | 159* | 117–210 | 26 | 100 | 65–147 | 75 | 132* | 103–166 |
| | 5–9 | 32 | 126 | 86–178 | 19 | 119 | 71–186 | 51 | 123 | 91–162 |
| | 10–14 | 31 | 156* | 105–221 | 19 | 116 | 69–181 | 50 | 138* | 102–182 |
| | 0–14 | 112 | 147* | 120–177 | 64 | 110 | 84–41 | 176 | 131* | 112–152 |

*95%CI for standardized incidence ratio (SIR) does not include 100.

and all diagnostic groups combined (SIR 147) in males. No significant differences were observed in south Asian females.

Standardized incidence ratios for the principal diagnostic groups by sex and 5-year age band are presented in Table 3. In the 0–4 age group, significant south Asian excesses were observed for males and both sexes combined for lymphoma (SIR 504 and 311) and all groups combined (SIR 159 and 132), and in the 10–14 age group for all groups combined (SIR 156 and 138). Again, there were no significant differences in south Asian females.

This study has identified a significant excess of childhood cancer among the south Asian population in England, predominantly due to higher rates in south Asian boys. It confirms previous reports, based on smaller regional data sets over longer periods, of an overall Asian excess (Powell et al, 1994) and of a higher proportion of male cases among Asians compared to non-Asians (Varghese et al, 1996). The pattern of type-specific occurrence in these data is also in keeping with previous studies, with higher south Asians rates reported for lymphoma (Powell et al, 1994; Varghese et al, 1996) and retinoblastoma (Parkes et al, 1994). This might be expected, as there is some overlap in the cases included in these studies, but approximately two-thirds of the cases in this series have not been previously reported and represent a larger population base. The finding of higher overall rates in south Asian children is in sharp contrast to the substantially lower overall cancer rate observed for south Asian adults compared to the non-south Asian adult population (Winter et al, 1999). Whilst sub-group variations may be important, they were not investigated because of insufficient numbers of cases and potential for misclassification.

While it is beyond the scope of this paper to attribute the excesses described in south Asian children to specific causes, the existing literature may provide clues to possible aetiologies. Large scale mixing of urban and rural populations has been associated with increases in childhood leukaemia and lymphoma (Kinlen et al, 1995). The higher rates of leukaemia in both male and female south Asian children could be construed as supporting this hypothesis, as it constitutes a significant excess in a migrant population largely originating from rural areas. Census estimates suggest that most of these cases will have been born in the UK but it is unlikely that their parents were (OPCS, 1993). The degree to which they can be regarded as an isolated population will be determined by their parents' level of contact with the dominant population. So genetic and other environmental causes must also be given due consideration. The striking excess of lymphoma among south Asian boys also requires explanation. The absence of a similar pattern for leukaemia argues against recording bias and it likely reflects an amplification of the excess seen in the general population.

Higher south Asian rates for hepatic tumours were observed in both children and adults (Winter et al, 1999). The link between hepatitis B infection and hepatocellular carcinoma is well established (Esteve et al, 1994) and a higher prevalence of hepatitis B infection has been reported in south Asian adults (Boxall et al, 1994). The contribution of hepatocellular carcinoma to the excess found in this dataset could not be established because histological coding was incomplete.

In the absence of self-ascribed ethnicity, attribution of south Asian ethnicity on the basis of names has been shown to be a feasible alternative (Nicoll et al, 1986; Cummins et al, 1999). With childhood cancers, the impact of misclassification resulting from the adoption of English names by south Asians is likely to be greater than for adult cancers. As the study protocol involved classifying doubtful names as non-south Asian, the consequence will have been to underestimate the true number of south Asian cases and misclassification of cases of mixed south Asian origin as south Asian will have been small. The excesses described therefore form minimal estimates. The broad consistency of the findings with those from previous studies using self-ascribed ethnicity to identify south Asian cases supports the validity of the methodology used.

Previous work suggests a transition towards higher western cancer risk in the migrant south Asian adult population (Winter et al, 1999) and this study confirms an excess cancer risk in south Asian children, the magnitude of which is probably of the order of 30–40% (Powell et al, 1994). The childhood excess is occurring in ethnic groups that display low cancer rates in the Indian subcontinent. While south Asians form a relatively small proportion of the

© 2001 Cancer Research Campaign 

total English population, approximately 50 per cent are under the age of 25 and the number of children at risk will therefore increase substantially over the next few decades. The higher overall rates and leukaemia and lymphoma incidence in south Asian children should be a cause for concern and explanations sought. Existing studies (UKCCS, 2000) may be informative in this regard, although a well-designed case–control study in the south Asian population will probably be required to address the key aetiological questions. Areas for investigation should include population mixing (Kinlen et al, 1995), paternal smoking (Sorahan et al, 1997), breast-feeding (Shu et al, 1999) and infant feeding patterns (Lawson et al, 1998).

### ACKNOWLEDGEMENTS

The authors would like to acknowledge the active contribution and collaboration of the following cancer registries: Thames Cancer Registry, Trent Cancer Registry, West Midlands Cancer Intelligence Unit, Yorkshire Cancer Registry. This study was supported by a grant from the Medical Research Council.

### REFERENCES

Boxall E, Skidmore S, Evans C and Nightingale S (1994) The prevalence of hepatitis B and C in an antenatal population of various ethnic origins. *Epidemiol Infection* **113**: 523–528

Bradford Health Authority. Nam Pehchan computer software (Version 1.1). New Mill, Victoria Road, Saltaire, Shipley BD18 3LD, UK. Computer Services, City of Bradford Metropolitan Council (Dept. 13), Britannia House, Bradford BD1 1HX, UK

Cummins C, Winter H, Cheng KK, Maric R, Silcocks P and Varghese C (1999) An assessment of the Nam Pehchan computer program for the identification of names of south Asian ethnic origin. *J Pub Health Med* **21**: 401–406

Esteve J, Benhamou E and Raymond L (1994) Descriptive Epidemiology. In: *Statistical Methods in Cancer Research*, vol 4. IARC Scientific Publications: number 128. *IARC*: Lyon

Kinlen LJ, Dickson M and Stiller CA (1995) Childhood leukaemia and non-Hodgkin's lymphoma near large rural construction sites, with a comparison with Sellafield nuclear site. *BMJ* **310**: 763–768

Kramarova E and Stiller CA (1996) The international classification of childhood cancer. *Int J Cancer* **68**: 759–765

Lawson MS, Thomas M and Hardiman A (1998) Iron status of Asian children aged 2 years living in England. *Arch Dis Child* **78**: 420–426

Mather HM, Chaturvedi N and Fuller JH (1998) Mortality and morbidity from diabetes in South Asians and Europeans: 11-year follow-up of the Southall Diabetes Survey, London UK. *Diabetic Med* **15**: 53–59

Muir KR, Parkes SE, Mann JR, Stevens MC and Cameron AH (1992) Childhood cancer in the West Midlands: incidence and survival, 1980–1984, in a multi-ethnic population. *Clin Oncol* **4**: 177–182

Muir KR, Parkes SE, Lawson S, Thomas AK, Cameron AH and Mann JR (1995) Changing incidence and geographical distribution of malignant paediatric germ cell tumours in the West Midlands Health Authority Region, 1957–92. *Br J Cancer* **72**: 219–223

Nicoll A, Bassett K and Ulijaszek SJ (1986) What's in a name? Accuracy of using surnames and forenames in ascribing Asian ethnic identity in English populations. *J Epidemiol Comm Health* **40**: 364–368

OPCS (Office of Population Censuses and Surveys) (1993) *1991 Census Report for England: Regional Health Authorities*. HMSO: London

Parkes SE, Amoaku WM, Muir KR, Willshaw HE and Mann JR (1994) Thirty years of retinoblastoma (1960–89): changing patterns of incidence. *Paediat Perinat Epidemiol* **8**: 282–291

Powell JE, Parkes SE, Cameron AH and Mann JR (1994) Is the risk of cancer increased in Asians living in the UK? *Arch Dis Child* **71**: 398–403

Powell JE, Kelly AM, Parkes SE, Cole TRP and Mann JR (1995) Cancer and congenital abnormalities in Asian children: a population-based study from the West Midlands. *Br J Cancer* **72**: 1563–1569

Shu XO, Linet MS, Steinbuch M, Wen WQ, Buckley JD, Neglia JP, Potter JD, Reaman GH and Robison LL (1999) Breast-feeding and risk of childhood acute leukemia. *J Natl Cancer Inst* **91**: 1765–1772

Soni Raleigh V, Bulusu L and Balarajan R (1990) Suicides among immigrants from the Indian subcontinent. *Br J Psychiat* **156**: 46–50

Sorahan T, Prior P, Lancashire RJ, Faux SP, Hulten MA, Peck IM and Stewart AM (1997) Childhood cancer and parental use of tobacco: deaths from 1971 to 1976. *Br J Cancer* **76**: 1525–1531

Stiller CA, McKinney PA, Bunch KJ, Bailey CC and Lewis IJ (1991) Childhood cancer and ethnic group in Britain: a United Kingdom Children's Cancer Study Group (UKCCSG) study. *Br J Cancer* **64**: 543–548

Swerdlow AJ, Marmot MG, Grulich AE and Head J (1995) Cancer mortality in Indian and British ethnic immigrants from the Indian subcontinent to England and Wales. *Br J Cancer* **72**: 1312–1319

UK Childhood Cancer Study Investigators (2000) The United Kingdom Childhood Cancer Study: objectives, materials and methods. *Br J Cancer* **82**: 1073–1102

Varghese C, Barrett JH, Johnston C, Shires M, Rider L and Forman D (1996) High risk of lymphomas in children of Asian origin: ethnicity or confounding by socioeconomic status? *Br J Cancer* **74**: 1503–1505

Wild S and McKeigue P (1997) Cross sectional analysis of mortality by country of birth in England and Wales, 1970–92. *Br Med J* **314**: 705–710

Winter H, Cheng KK, Cummins C, Maric R, Silcocks P and Varghese C (1999) Cancer incidence in the south Asian population of England (1990–92). *Br J Cancer* **79**: 645–654

*© 2001 Cancer Research Campaign*

